# Molecular Survey of Chlamydial Infections in Three Public Bird Collections in Tehran, Iran

**DOI:** 10.1002/vms3.70537

**Published:** 2025-08-15

**Authors:** Seyed Mohamad Mahdi Hashemian, Seyed Ahmad Madani, Seyed Mostafa Peighambari

**Affiliations:** ^1^ Central Veterinary Laboratory Tehran Iran; ^2^ Department of Animal and Poultry Health and Nutrition Faculty of Veterinary Medicine University of Tehran Tehran Iran; ^3^ Department of Avian Diseases Faculty of Veterinary Medicine University of Tehran Tehran Iran

**Keywords:** avian chlamydiosis, *Chlamydia*, *Chlamydia psittaci*, zoo, zoonoses

## Abstract

**Background:**

Avian *Chlamydia* spp. are capable of infecting different avian species and potentially cause the loss of valuable birds in rehabilitation facilities and zoos. They also pose a potential zoonotic risk to visitors and workers at such centres.

**Objectives:**

This study aimed to assess the occurrence of chlamydia in two different public aviaries and a rehabilitation centre.

**Method:**

One hundred and eight samples from 48 different avian species belonging to 11 different orders were collected. These samples were tested for chlamydia infection by detecting the *Chlamydia 16s rRNA* gene using polymerase chain reaction.

**Results:**

Thirty‐seven samples were positive for *Chlamydia* DNA. High infection rates were detected in Psittaciformes (60%) and Columbiformes (77.8%). These findings indicate the relatively high frequency of chlamydial infections in birds of these orders. The occurrence of this infection in Falconiformes was 33.3%. Galliformes species investigated in this study had a lower occurrence (16.7%) of chlamydial infection. The only sample taken from the Charadriiformes order belonging to the yellow‐footed gull was tested positive. A relatively high rate of infection with chlamydial agents was demonstrated in this study.

**Conclusion:**

Regarding the close contact of infected animals with both workers and visitors, these findings are alarming. The affected aviary centres must implement a strategy to monitor, detect and control the infection, as it poses a considerable public health risk. On the other hand, the infection of rehabilitating captive birds in rescue centres is particularly relevant because the infection might be reintroduced to endangered wild populations, posing a conservation and environmental hazard.

## Graphical abstract

One hundred and eight samples from different avian species were collected and tested for *Chlamydia* spp. infection using PCR. Thirty‐seven samples from Psittaciformes, Columbiformes, Falconiformes, Galliformes and Charadriiformes were positive for *Chlamydia* DNA. A relatively high rate of infection was demonstrated in three zoological collections in Tehran, Iran.

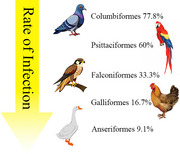

## 1 Introduction

Chlamydiosis is a disease caused by a group of gram‐negative, intracellular bacteria belonging to the Chlamydiaceae family. Within the genus *Chlamydia* there are currently at least 15 known species, including *C. abortus*, *C. avium*, *C. caviae*, *C. felis*, *C. gallinacea*, *C. muridarum*, *C. pecorum*, *C. pneumoniae*, *C. psittaci*, *C. suis, C. trachomatis, C. buteonis, C. crocodili, C. serpentis* and *C. poikilothermis*, in addition to four proposed species: *C. corallus, C. ibidis, C. sanzinia* and *C. testudinis* (Chaiwattanarungruengpaisan et al. 2021; Kuo et al. 2015; Laroucau et al. 2020, 2019; Sachse et al. 2015; Staub et al. 2018; Taylor‐Brown et al. 2016; Vorimore et al. 2013). The infection with Chlamydiaceae has been reported in more than 460 avian species from 30 different orders (Kaleta and Taday 2003). In addition to *C. psittaci*, *C. gallinacea*, *C. avium*, *C. buteonis* and *C. ibidis* have also been identified in avian species (Laroucau et al. 2019; Sachse et al. 2014; Vorimore et al. 2013). Outcome of *C. psittaci* infection in birds can vary from a subclinical infection with no apparent clinical signs to a severe systematic disease affecting different organs, causing mortality in affected birds (Vanrompay 2020). The infective elementary bodies of *C. psittaci* are excreted in faeces and other body fluids from the infected host, and others can be infected either orally or by inhalation (Harkinezhad et al. 2009). The infection can persist for months in avian species. Affected birds, particularly those with subclinical forms, can shed the bacteria intermittently. Stress factors, such as crowding, weather changes, transportation and concurrent diseases, can initiate shedding in chronically contaminated birds (Harkinezhad et al. 2009; Vanrompay 2020).

The zoonotic potential of *C. psittaci* in humans and its possible transmission from different avian species to other mammalian hosts highlighted the epidemiologic significance of this important agent (Sachse et al. 2015). Humans can be infected by inhaling contaminated particles from avian droppings or dried discharges from shedding birds (Telfer et al. 2005; Williams et al. 1998). The disease in humans was historically called psittacosis or parrot fever (Vanrompay 2020). The human infection can also vary from a subclinical disease to a severe atypical pneumonia, and it can occasionally be fatal. The man‐to‐man transmission of *C. psittaci* has rarely been reported (Wallensten et al. 2014). All chlamydia agents are potentially zoonotic, but *C. psittaci* and *C. abortus* infections have been more frequently reported (Rodolakis and Yousef Mohamad 2010).

Chlamydia infection in pet birds in Iran has been extensively reported in recent years (Abbasi et al. 2019; Madani et al. 2011; Madani and Peighambari 2013; Mahzounieh et al. 2020; MoradiSarmeidani et al. 2020; Razmyar et al. 2016). There are also numerous reports of this infection in feral and captive pigeons (Doosti and Arshi 2011; Ghorbanpoor et al. 2015; Golestani et al. 2020; Madani and Peighambari 2013). On the other hand, there are few reports of chlamydial infections in commercial (Hashemian et al. 2023; Tatari et al. 2016) and wild birds in Iran (Mahzounieh et al. 2020). The possible transmission of *Chlamydia* to bird owners and fanciers who have close contact with infected animals has also been demonstrated in recent studies in Iran (Mahzounieh et al. 2020; MoradiSarmeidani et al. 2020). Despite numerous reports of avian chlamydiosis in ornithological gardens and rehabilitation centres worldwide (Aaziz et al. 2015; Matsui et al. 2008; Raso et al. 2002), no studies have attempted to detect chlamydial infection in public zoos and bird collections in Iran.

The purpose of this study was to assess the occurrence of chlamydial infections in birds kept in public zoological gardens. In this study, a polymerase chain reaction (PCR) test was applied to detect chlamydial infections in three different ornithological collections in Tehran. Chlamydial infection in these collections may pose a zoonotic risk to zoo workers, aviary veterinarians and regular visitors of these zoological gardens.

## 2 Materials and Methods

### 2.1 Sampling

Clinical specimens were collected from three public ornithological collections in Tehran. Collection A was an amusement park featuring both mixed‐species and single‐species exhibition aviaries. Collection B was a wildlife rehabilitation centre with some exhibition aviaries for public visit. Collection C was multispecies zoological garden with special cages and aviaries for bird exhibition. When possible, triple swab samples from the conjunctiva, choanal cleft and cloaca, respectively, were collected individually from each bird with a single swab as it was formerly described by Madani et al. (2013). In large aviaries when capture and individual sampling was not possible, pooled droppings were collected. Swab samples were placed in 2 mL microtubes containing 1 mL of sucrose‐phosphate‐glutamate (SPG) transport medium (Warford et al. 1984).

### 2.2 Polymerase Chain Reaction

DNA was extracted from at least 200 µL of SPG using MBST extraction kit (Molecular Biological System Transfer, Tehran, Iran) following the manufacturer's instructions. A robust PCR targeting the conserved region of the *16s rRNA* gene using 16S‐IGF (5′‐GAT GAG GCA TGC AAG TCG AAC G‐3′) and 16S‐IGR (5′‐CCA GTG TTG GCG GTC AAT CTC TC‐3′) primers was conducted. A 298‐bp product specific to the order Chlamydiales was amplified (Borel et al. 2006). The PCR reaction mix consisted of 2.5 µL of PCR buffer, 1 µM of each primer, 200 µM dNTPs, 3500 µM of MgCl_2_, 0.2 µL *Taq* DNA polymerase (all from Sinaclon, Tehran, Iran) and 17.3 µL of distilled deionized water. A volume of 2.5 µL of extracted DNA was added to the reaction mixture. DNA amplification was then initiated with 15 min of 95°C and continued with 45 cycles of denaturation at 94°C for 30 s, annealing at 51°C for 30 s and elongation at 72°C for 45 s with a final extension step for 5 min at 72°C (Zweifel et al. 2009). PCR products were illuminated by 1.5% agarose gel electrophoresis containing 0.004% RedSafe (Intron Biotechnology, Seongnam, South Korea) under an ultraviolet transilluminator. The positive control DNA template used in this study was extracted from the *C. psittaci* strain UT‐245, which was detected in an African grey parrot (*Psittacus erithacus*) (Madani and Peighambari 2013).

## 3 Results

A total of 108 samples from 48 different species belonging to 11 different avian orders were collected from the bird collections. Thirty‐seven samples (34.26%) were positive for *Chlamydia* spp. DNA using PCR.

Twenty‐three of 55 samples (41.8%), seven of 31 samples (22.6%) and seven of 22 samples (31.8%) were positive for *Chlamydia* spp. DNA in collections A, B and C, respectively.

The infection rates among the 11 avian orders surveyed in this investigation are summarized in Table 1. The infection rates were highest among Columbiformes (77.8%) and Psittaciformes (60%). The only triple‐swab sample, which was collected individually from a yellow‐legged gull (*Larus michahellis*), was positive. In addition, samples from golden pheasant (*Chrysolophus pictus*), domestic chicken (*Gallus gallus domesticus*), domestic goose (*Anser anser domesticus*), griffon vulture (*Gyps fulvus*), Egyptian vulture (*Neophron percnopterus*), northern goshawk (*Accipiter gentilis*), saker falcon (*Falco cherrug)*, Eurasian hobby (*Falco subbuteo*), common buzzard (*Buteo buteo*), red kite (*Milvus milvus*), golden eagle (*Aquila chrysaetos*) and steppe eagle (*Aquila nipalensis*) were positive. Five pooled droppings samples taken from a mixed species cage containing budgerigars (*Melopsittacus undulatus*) and zebra finches (*Taeniopygia guttata*) were all positive. Samples from Passeriformes, including common mynas (*Acridotheres tristis*), a western jackdaw (*Coloeus monedula*) and red‐billed choughs (*Pyrrhocorax pyrrhocorax*), were negative. There are also no *Chlamydia*‐positive samples in specimens collected from three grey crowned cranes (*Balearica regulorum*), a greater flamingo (*Phoenicopterus roseus*), a great white pelican (*Pelecanus onocrotalus*) and three Eurasian eagle‐owls (*Bubo bubo*) in these three bird collections.

**TABLE 1:** Frequency of positive samples for the presence of *16S rRNA* DNA of *Chlamydia* sp. in different avian orders in three public bird collections in Tehran, Iran.

Avian order	Bird collection	Number of samples	Positive (%)	Total no. (%)	Total positive (%)
Psittaciformes	A	12	7 (58.3)	15	9 (60)
	B	0	—		
	C	3	2 (66.6)		
Columbiformes	A	9	7 (77.8)	9	7 (77.8)
	B	0	—		
	C	0	—		
Gruiformes	A	0	—	3	0
	B	0	—		
	C	3	0		
Pelecaniformes	A	0	—	1	0
	B	0	—		
	C	1	0		
Phoenicopteriformes	A	0	—	1	0
	B	0	—		
	C	1	0		
Galliformes	A	19	3 (15.8	24	4 (16.7)
	B	4	0		
	C	1	1 (100)		
Anseriformes	A	7	1 (14.3)	11	1 (9.1)
	B	1	0		
	C	3	0		
Passeriformes	A	3	0	5	0
	B	1	0		
	C	1	0		
Falconiformes	A	0	—	30	10 (33.3)
	B	22	6 (27.3)		
	C	8	4 (50.0)		
Strigiformes	A	0	—	3	0
	B	2	0		
	C	1	0		
Charadriiformes	A	0	—	1	1 (100)
	B	1	1 (100)		
	C	0	—		
Mixed species (Finches and Budgerigars)	A	5	5	5	5 (100)
	B	—	—		
	C	—	—		
	Total			108	37 (34.26)

Regarding the different sampling methods, 14 out of 48 (29.2%) triple swab specimens, which were individually collected after capture, were positive. Twenty‐three out of 60 (38.3%) pooled droppings samples were positive for *Chlamydia* DNA.

## 4 Discussion

In this study, chlamydial infection in three public bird collections in Tehran was investigated using PCR targeting the *16s rRNA* gene. All three collections were positive, and the infection rate was relatively high (34.3%). Chlamydial infections have been reported in six of 11 avian orders. Samples from 48 different avian species were positive for chlamydial DNA. Regarding the zoonotic potential of the avian *Chlamydia* spp., particularly *C. psittaci*, these findings might raise public health concerns regarding the zoological gardens. The identification and characterization of *Chlamydia* species was beyond the scope of the present study, but a bulk of previous studies revealed the higher prevalence of *C. psittaci* in avian species (Golestani et al. 2020; Kalmar et al. 2014; Mattmann et al. 2019; Sareyyupoglu et al. 2007; Stalder et al. 2020). In addition to the known avian specific species, strains of *C. abortus* have also been detected in birds. These strains might be zoonotic and pathogenic in humans (Origlia et al. 2019; Raven et al. 2024; Szymańska‐Czerwińska et al. 2017).

The set of primers that was used in this study (16S‐IGF/16S‐IGR) was previously used to detect *Chlamydia*‐positive birds among raptor and crows which their samples were negative in two other species‐specific conventional PCRs for detecting *C. psittaci and C. buteonis*. According to that study, this conventional PCR has an acceptable sensitivity for detecting *C. psittaci* DNA (Stalder et al. 2020). The same primers have also been used to detect other *Chlamydia* species, such as *C. pneumoniae*, *C. suis* and *C. abortus*, and are considered order specific primers (Blumer et al. 2007). In a study, positive samples from pigeons, song birds and waterfowl that could not be identified using the ArrayTube microarray assay were further investigated using these primers for species determination or genotyping (Zweifel et al. 2009). Borel et al. (2008) identified six different species of chlamydia, including *C. psittaci*, from a bird using these primers. A comparison of serology (CFT), fluorescent antibody test, two different staining methods and PCR with the same primers showed that this PCR method was the most sensitive. In that study 96.4% of samples from wild hoopoe and 90.6% of samples collected from wild cattle egrets were positive by PCR (El‐Jakee et al. 2014).

A high percentage of samples taken from pigeons were positive (77.8%), highlighting the importance of pigeons in the epidemiology of chlamydial infections. Studies on feral pigeons have revealed that these birds are important carriers of zoonotic agents and are capable of transmitting almost 110 pathogens to humans, including eight viruses, 41 bacteria, 55 fungi and six protozoa (Magnino et al. 2009). *C. avium* and *C. psittaci* were the primary chlamydial species found in pigeons in different studies (Burt et al. 2018; Golestani et al. 2020; Kik et al. 2020; Mattmann et al. 2019). *C. psittaci* is one of the most important zoonotic agents in these birds (Haag‐Wackernagel and Moch 2004; Magnino et al. 2009). The results of the present study are in accordance with those of other studies showing high infection rates in pigeons (Donati et al. 2015; Golestani et al. 2020; Magnino et al. 2009; Mattmann et al. 2019). Infected pigeons are usually asymptomatic carriers of *C. psittaci*, which increases the chance of transmission to people and other animals. Shedding of the organism occurs intermittently and probably in response to stressors such as crowding, breeding or concurrent infections (Harkinezhad et al. 2009).

In this investigation, a relatively high prevalence of the infection was also found in Psittaciformes (60%). Psittacine birds are believed to be the primary hosts of *C. psittaci*. Numerous reports have assessed the prevalence of *Chlamydia* infection in captive or free‐living parrots. In a study in Costa Rica, only 3.4% of captive parrots from clinics or wild life rescue centres showed positive results (Sheleby‐Elías et al. 2013). In Poland, 10.3% of captive parrots in captivity from aviaries and zoological shops were positive by PCR (Piasecki et al. 2012). In a study in Brazil, using serology for antibody detection and antigen detection methods, 44% of birds had active shedding of *Chlamydia* DNA, whereas 77.3% of birds were seropositive (Raso et al. 2002). In Australia, 9.8% of free‐flying parrots were positive for *C. psittaci* DNA and seroprevalence rate of this infection was 37%. In psittacine breeding centres in Belgium, 39 facilities and 46 human samples were investigated. Thirteen centres (33.3%) were positive in PCR, but *C. psittaci* was isolated from 20.5%. Six human samples were positive using PCR, but the bacteria could be isolated from four specimens in cell culture propagation (Vanrompay et al. 2007). In another study in Belgium, five African grey parrots (*P. erithacus*) in a breeding centre, along with the specimens from the owner, a veterinarian and an assistant, were *C. psittaci*‐positive as well (Harkinezhad et al. 2007). The latter study demonstrated the zoonotic significance of chlamydial infection in parrot collections. Parrots and pigeons are the primary hosts for *C. avium* that can cause both subclinical (Origlia et al. 2023) and fatal infection in these birds (Popelin‐Wedlarski et al. 2020).

In our study, 10 raptors from the order Falconiformes (33.3%) belonging to nine different species were positive. In Sweden, 319 raptors were sampled and examined for *C. psittaci*. Two peregrine falcons (*Falco peregrinus*) (1.9%) and two white‐tailed sea eagles (*Haliaeetus albicilla*) (1%) were positive (Blomqvist et al. 2012). In California, *C. psittaci* was isolated from four dead red‐tailed hawks (*Buteo jamaicensis*) and seven other birds in the same facility in 1983. In a subsequent investigation, 100 raptors from 14 different species were examined. The organism was isolated from almost 10% of them, and 44% of the blood samples were positive for the antibody against *C. psittaci* (Fowler et al. 1990). In a study conducted in Switzerland, 20 of 341 raptors (5.9%) were positive for *Chlamydia* DNA (Stalder et al. 2020). In Germany, 39 samples from free‐living raptors were investigated, and 74% of the birds were positive in PCR (Schettler et al. 2003). In a raptor rehabilitation centre in Spain, 54 asymptomatic free‐living raptors were sampled upon arrival, and 40.6% were positive (Ortega et al. 2012). *C. buteonis* is a novel species of *Chlamydia* discovered by Laroucau et al. (2019) in a red‐shouldered hawk. Other studies in the United States of America and the United Arab Emirates showed that this organism is prevalent in both wild (Hawkins et al. 2025; Seibert et al. 2021) and captive raptor populations (Vorimore et al. 2024).

Four samples (16.7%) from Galliformes, including three golden pheasants (*C. pictus*) and one chicken, were positive for the presence of the *Chlamydia 16s rRNA* gene. In Poland, three pheasants from a small collection of seven birds harboured *C. psittaci* DNA (Krawiec et al. 2015). In a study in the Slovak Republic, up to 40.4% of pheasants were seropositive using the complement fixation test (Trávnicek et al. 2002). Chickens were used to be considered relatively resistant to chlamydial infection, but recently, a new chlamydial agent named *C. gallinacea* was shown to be prevalent in chickens (Guo et al. 2016; Li et al. 2017; Marchino et al. 2022; Ornelas‐Eusebio et al. 2020; Sachse and Laroucau 2014).

The only sample from the order Charadriiformes that was a yellow‐legged gull was positive. Further investigations are required to elaborate the rate of the infection in sea birds of Iran. Former studies revealed that the prevalence of chlamydial infection in sea birds was 18.5% in France (Aaziz et al. 2015) and 8% in Bering Sea (Christerson et al. 2010). The detected agents were mostly un‐classified or atypical *Chlamydia* spp. However, the detected atypical agents in cloacal swabs or faecal specimens of sea birds could be primarily related to fish or amoeba that were consumed by birds (Isaksson et al. 2015).

A sample from a domestic goose was positive, out of 11 samples taken from Anseriformes. As domestic goose is extensively held by villagers in Iran, having chlamydiosis in mind as a possible respiratory infection is important for veterinarians. In a study in Belgium, 93.8% of asymptomatic Canada geese were seropositive using ELISA and *C. psittaci* was isolated from 58% of them (Dickx et al. 2013). All samples taken from orders Gruiformes (three grey crowned cranes), Pelecaniformes (one great white pelican), Phoenicopteriformes (one greater flamingo) and Strigiformes (three Eurasian eagle owl) were negative.

The only rehabilitation centre investigated in this study showed a significant occurrence of the infection, especially in raptors. This finding is even more significant regarding the many valuable animals that are kept at this wildlife rescue centre. Overcrowding in enclosures was evident in this centre, and therefore, the transmission of infectious agents is facilitated. Wild‐caught rescued birds could be the source of infection. On the other hand, the transmission within the facility was also possible regarding the close contact and high stocking density of the cages. Execution of hard biosecurity measurement, particularly a strict quarantine strategy before introduction of new birds into the facility, seems necessary. Sampling and monitoring of submitted animals for chlamydial infection will aid to prevent the exposure of rescued animals.

In a zoological park in New Zealand, three veterinary staff members had *C. psittaci* antibodies (Forsyth et al. 2012). Pharyngeal samples of 3 of 10 staff workers at an avian refugee centre were positive for viable *C. psittaci* culture (Kalmar et al. 2014). As *C. psittaci* is an agent of zoonotic importance, monitoring birds in such places is reasonable. The two other investigated facilities in this survey had a remarkable number of daily visitors. In a study on zoo animals in Japan, *C. psittaci* was confirmed in 7.2% of birds kept in five different zoos (Kabeya et al. 2015). In another study, *C. psittaci* was found in 36.5% of parrots at different zoos, parrot farms and exhibitions (Lee et al. 2023).

## 5 Conclusion

This study aimed to determine the occurrence of chlamydial infections in three aviaries in Tehran, Iran. However, the species of *Chlamydia* was not identified in the present study. The outstanding infection rate in these centres poses a high risk of possible zoonoses in both workers and visitors. Regarding the close contact of the infected animals with both workers and visitors, these findings are alarming. The affected aviary centres must implement a strategy to monitor, detect and control the infection, as it poses a considerable public health risk. On the other hand, the infection of rehabilitating captive birds in rescue centres is particularly relevant because the infection might be reintroduced to endangered wild populations, posing a conservation and environmental hazard. Due to funding limitations, DNA sequencing of positive samples was not performed in this study. Further studies are required to identify and characterize chlamydial agents in these centres.

## Author Contributions

Study concept and design: Seyed Ahmad Madani and Seyed Mostafa Peighambari. Acquisition of data: Seyed Mohamad Mahdi Hashemian. Analysis and interpretation of data: Seyed Ahmad Madani. Drafting of the manuscript: Seyed Mohamad Mahdi Hashemian and Seyed Ahmad Madani. Critical revision of the manuscript for important intellectual content: Seyed Ahmad Madani and Seyed Mostafa Peighambari. Study supervision: Seyed Ahmad Madani and Seyed Mostafa Peighambari.

## Ethics Statement

The sampling and the other interventions concerning with live animals were approved ethically by the research committee of the Faculty of Veterinary Medicine, University of Tehran.

## Conflicts of Interest

The authors declare no conflicts of interest.

## Peer Review

The peer review history for this article is available at https://www.webofscience.com/api/gateway/wos/peer‐review/10.1002/vms3.70537.

## Data Availability

The data that support the findings of this study are available from the corresponding author, [Seyed Ahmad Madani], upon reasonable request.
